# Percutaneous nephrolithotomy versus open surgery for surgical treatment of patients with staghorn stones: A systematic review and meta-analysis

**DOI:** 10.1371/journal.pone.0206810

**Published:** 2019-01-31

**Authors:** Yiwen Chen, Jianhua Feng, Haifeng Duan, Youwei Yue, Chaofeng Zhang, Tuo Deng, Guohua Zeng

**Affiliations:** 1 Department of Urology, Minimally Invasive Surgery center, The First Affiliated Hospital of Guangzhou Medical University, Guangzhou, Guangdong, China; 2 Department of Urology, Longgang District Central Hospital, Shenzhen, Guangdong, China; Tongji Hospital, Tongji Medical College, Huazhong University of Science and Technology, CHINA

## Abstract

**Objectives:**

To compare the efficacy and safety of percutaneous nephrolithotomy (PCNL) and open surgery (OS) for surgical treatment of patients with staghorn stones based on published literatures.

**Materials and methods:**

A comprehensive literature search of Pubmed, Embase, CNKI and Cochrane Library was conducted to identify studies comparing outcomes of PCNL and OS for treating patients with staghorn stones up to Jan 2018.

**Results:**

There was no significant difference in final-SFR between PCNL and OS (odds ratio[*OR*]: 1.17; 95% confidence interval [*CI*]: 0.64, 2.15; *p* = 0.61), while PCNL provided a significantly lower immediate-SFR compared with OS (*OR*: 0.29; 95% *CI*: 0.16, 0.51; *P* < 0.0001). PCNL provided significantly lower overall complication rate, shorter operative times, hospitalization times, less blood loss and blood transfusion compared with OS (*OR*: 0.59; 95% *CI*: 0.41, 0.84; *P* = 0.004), (weighted mean difference [*WMD*]: -59.01mins; 95% *CI*: -81.09, -36.93; *p* < 0.00001), (*WMD*: -5.77*days*; 95% *CI*: -7.80, -3.74; *p* < 0.00001), (*WMD*: -138.29*ml*; 95% *CI*: -244.98, -31.6; *p* = 0.01) and (*OR*: 0.44; 95% *CI*: 0.29, 0.68; *P* = 0.00002), respectively. No significant differences were found in minor complications (Clavien I-II) (*OR*: 0.72; 95% *CI*: 0.47, 1.09; *p* = 0.12) and major complications (Clavien III-V) (*OR*: 0.5; 95% *CI*: 0.23, 1.08; *P* = 0.08). In subgroup analysis, there were no significant differences for overall complications and operative times between mini-PCNL and OS. In sensitivity analysis, there was no significant difference for overall complications between PCNL and OS.

**Conclusion:**

Our analysis suggested that standard PCNL turns out to be a safe and feasible alternative for patients with staghorn stones compared to OS or mini-PCNL. Because of the inherent limitations of the included studies, further large sample, prospective, multi-centric and randomized control trials should be undertaken to confirm our findings.

## Introduction

Staghorn stones still represent an intractable challenge to urologists. Open surgery (OS) was once considered to be the “gold standard” for the surgical treatment of staghorn calculi. However, thanks to miniaturization of endoscopic devices, increasing quality of optic systems, advent of holmium laser use and increasing experience in endoscopic surgery, the surgical management of staghorn calculi has been revolutionized. And the American Urological Association(AUA) guidelines for the management of staghorn calculi recommend percutaneous nephrolithotomy (PCNL) as the modality of choice and standard of practice[1].

However, not only the stone burden but also the morphology of stones can significantly affect the outcomes of PCNL in the management of staghorn calculi[2,3]. This is in contrast to OS, which is little affected by the morphometric index of staghorn calculi. Due to some reasons such as the unavailability of surgical instruments, higher stone free rate(SFR) or shorter operative times, many urologists still recommend OS for patients with complex staghorn calculi[4–6].

Some previous studies[6–15] had compared PCNL versus OS. Nevertheless, all these studies were small samples, and the results were controversial and inconclusive. The optimal treatment for staghorn calculi is still under debate. No meta-analysis has investigated the efficacy and safety of those two procedures. And whether PCNL is safer or more effective when compared to the OS remains unsettled. Therefore, to provide comprehensive information about the strategy of PCNL as well as OS in the treatment of staghorn renal stones, we performed this systematic review and meta-analysis of published studies comparing the efficacy and safety of PCNL and OS for surgical treatment of patients with staghorn renal stones. We hope it may guide urologists and patients to decide on the treatment modality, and to select the optimal treatment.

## Materials and methods

A prospective protocol of objectives, literature-search strategy, inclusion and exclusion criteria, study selection, data extraction, outcome measurements, and methods of statistical analysis was prepared a priori according to the Preferred Reporting Items for Systematic Reviews and Meta-analysis[16].

### Search strategy

A comprehensive literature strategy search was performed by two members (Chen and Zhang) independently in Jan 2018. The PubMed, Embase, CNKI and the Cochrane Library databases were used to identify relevant studies up to Jan 2018. Separate searches were done with the following search terms: “‘percutaneous nephrolithotomy’ or ‘PCNL’ or ‘PCN’” and “‘Open surgery’ or ‘OS’” in combine with “‘staghorn calculi’ or ‘staghorn renal stones’”.

### Inclusion and exclusion criteria

The selected studies were included based on the following criteria: (1) studies reported comparison between PCNL and OS in patients with staghorn calculi; (2) the outcome measures consisted of at least two of the following things: complications, SFR, hospitalization times, operative times, blood loss, and blood transfusion. Exclusion criteria are as follows: (1) repeated publications or conference proceedings; (2) non-published materials, editorials or reviews; (3) studies containing patients with serious urinary infection, renal insufficiency, musculoskeletal deformities, solitary kidney or congenital abnormalities.

### Study selection and data extraction

We screened the studies according to inclusion and exclusion criteria. Two authors (Feng and Yue) independently extracted data and appraised both quality and content. We contacted the authors of relevant studies to supplement incomplete data. Where disagreement arose, papers were re-examined and discussed, and the consensus was reached by the adjudicating senior authors (Zeng and Chen). The extracted data including: first author, year of publication, baseline patient characteristics, study period, study design, interventions, outcome measures, variations in PCNL techniques, statistical methods, and study conclusions. The outcomes included complications, SFR, hospitalization times, operative times, blood drop, and blood transfusion.

### Quality assessment and statistical analysis

The level of evidence (LE) of all included studies was assessed by the criteria provided by the Oxford Centre for Evidence-based Medicine[17]. And the quality of non-randomized controlled trials (non-RCTs) was assessed by Newcastle-Ottawa Scale.[18] The Cochrane risk of bias tool was applied to assess the methodological quality of RCTs.[19] All the meta-analyses were performed using Review Manager 5.2 software. The odds ratio (OR) and weighted mean difference (WMD) were used to compare dichotomous and continuous variables, respectively. All results were reported with 95% confidence intervals (CIs). Chi-square test and I-square test were used for testing heterogeneity between studies. If heterogeneity was not significant (*P* > 0.10, *I*^*2*^ < 50%), fixed-effect model was employed, otherwise, random-effect will be adopted. The results of the meta-analysis were expressed using forest graphs or tables. The Z-test determined the pooled effects, and *P* < 0.05 was considered statistically significant. Subgroup analyses were performed to compare standard PCNL and mini-PCNL with OS. Funnel plots were used to screen for potential publication bias. Sensitivity analysis was undertaken using studies of high quality. Only outcomes with three or more than three studies were included in the sensitivity analysis.

## Results

**Fig 1:**
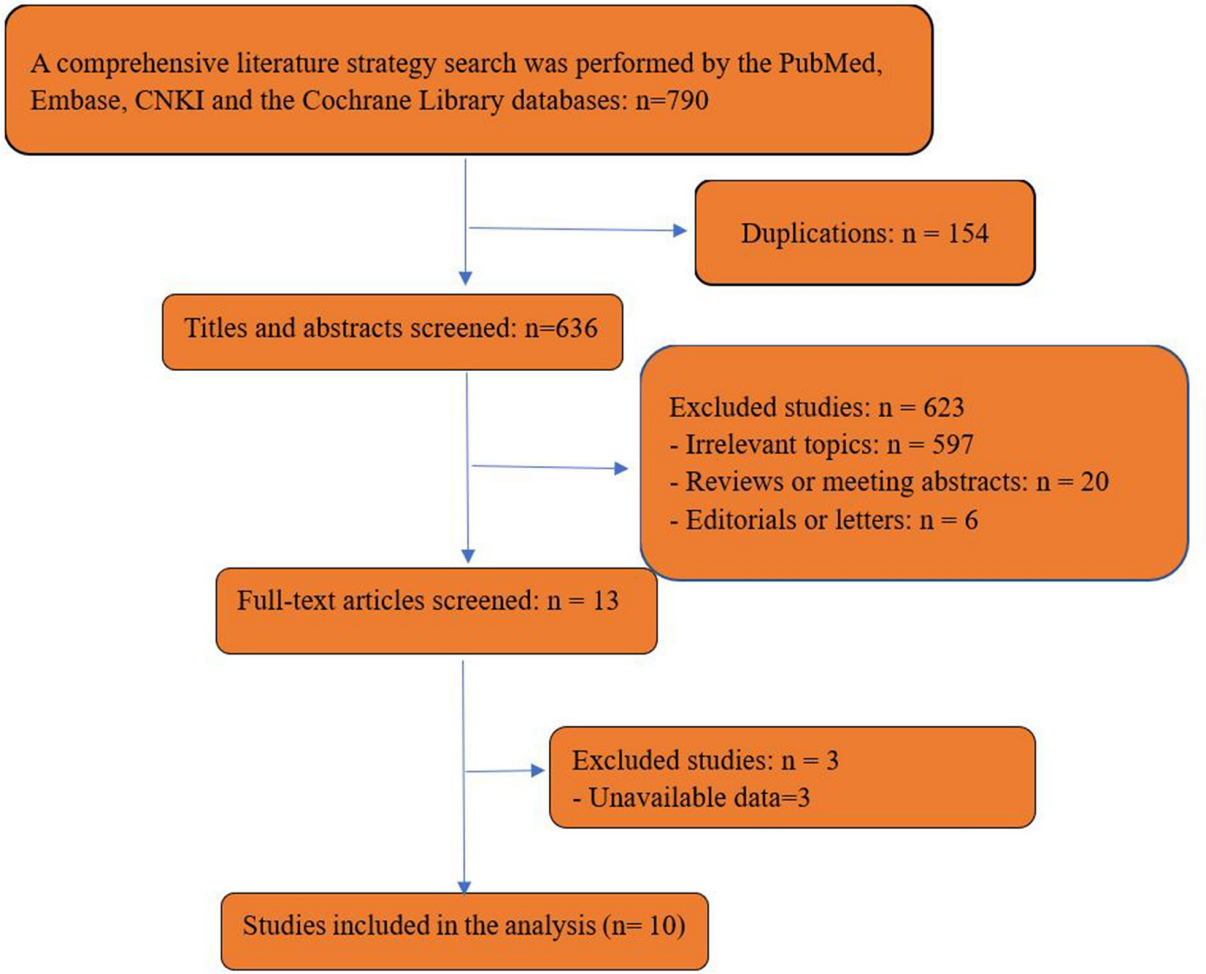
Flow diagram of studies identified, included, and excluded.

The search strategy generated 790 studies. After an initial screening of title and abstract, 13 studies were thought to meet the inclusion criteria. After further screening of full text, we excluded 3 articles because of unavailable data. 10 studies[6–15], which included 921 patients (531 cases for PCNL and 390 cases for OS) fulfilled the predefined inclusion criteria, and were included in the final analysis (Fig 1). Examination of the references listed for these studies and for the review articles did not yield any further studies for evaluation. Table 1 shows the baseline characteristics and quality assessment of all included studies. Among 10 included studies available for meta-analysis, 2 were prospective case-control studies[8,15] (LE: 3b), 7 were retrospective case-control studies[6,9–14] (LE: 3b) and 1 was RCT[7] (LE: 2b). The methodological qualities of included studies were relatively high for six of the nine non-randomized studies[6,8–10,14,15] (NOS: 6 of 9 points), whereas three studies[11–13] were moderate quality with 5 scores. And the only one RCT[7] was high quality for 5 points (the Cochrane risk of bias tools: score from 0 to 7).

**Table 1:** The baseline characteristics and quality assessment of all included studies.

Study	country	Study period	Study design	LE	Mean age ± SD (years)		Mean stone size		Gender(male/female)		cases, n		Study quality
					PCNL	OS	PCNL	OS	PCNL	OS	PCNL	OS	
AL-KOHLANY KM et al 2005	Egypt	2001–2003	RCT	2b	48.6±8.5	48.7±10.9	18.7±6.9 cm^3^	18.8±8.1 cm^3^	17/26	23/22	43	45	5#
Aminsharifi A et al 2016	Iran	2010–2015	PCCS	3b	48±8.57	48.21±7.87	79.06 ± 15.63 mm	77.0 ± 14.33 mm	13/3	11/3	16	14	7*
El-Nahas AR et al 2014	Egypt	2000–2013	RCCS	3b	7.1±2.93	7.6±3.31					28	28	6*
Falahatkar S et al 2009	Iran	2005–2006	PCCS	3b	46.5±13.4	46.04±13.6			35/37	19/29	72	48	6*
Zhang FBY et al 2017	Taiwan	2007–2013	RCCS	3b	54.3±411.6	50.5±11.1	19.8±5.6 cm^2^	19.7±6.4 cm^2^	21/40	3/8	61	11	7*
Cao GZ et al 2008	China	2003–2007	RCCS	3b	43.00±12.57	45.00±10.36	38.38 ±7.85cm	40.04 ±9.64cm	14/46	12/36	60	48	6*
Fei X et al 2012	China	2003–2011	RCCS	3b	46.5±14.5	42.3 ±10.5	3.7±1.6 cm^2^	3.5±1.4cm^2^			54	48	5*
liang TS et al 2010	China	2003–2008	RCCS	3b	46.37±14.75	48.35±14.13	3.17±1.8cm	2.94±1.50cm	47/32	24/17	79	41	6*
Yang X et al 2014	China	2010–2012	RCCS	3b	45.1±3.2	43.6±3.8	37.9±2.5mm	36.7±2.6mm	26/20	25/15	46	40	5*
Zheng B et al 2011	China	2007–2010	RCCS	3b	45.56±10.23	44.32±11.11	41.01±7.30mm	42.34±6.96mm	39/33	39/31	72	70	5*

LE = level of evidence, PCNL = Percutaneous nephrolithotomy, OS = open surgery, PCCS = prospective case controlled study, RCCS = retrospective case controlled study, RCT = randomized controlled trial.

* Using Newcastle-Ottawa Scale (score from 0 to 9). # Using The Cochrance collaboration's tool (score from 0 to 7).

Surgical technique for PCNL among all including studies varied in terms of image guidance, type of dilator, sheath size, type of lithotripsy, postoperative stent, and postoperative nephrostomy tube (Table 2). In 4 studies[8,9,11,15], percutaneous accesses were achieved under fluoroscopic guidance, 4 studies[6,12–14] under ultrasound, 1 study[10] were combined fluoroscopic guidance with ultrasound and one study[7] was not recorded. Tract dilation was accomplished using Amplatz dilators in 7 studies[6,8–11,14,15]. One studies[12] used Metal dilators. And one study[7] was not recorded. Three studies[10,11,14] were mini-PCNL with sheath sizes less than 24Fr, while 7 studies[6–9,12,13,15] were standard PCNL with sheath sizes greater than or equal to 24Fr. Fragmentation and stone removal was accomplished by pneumatic energy in 9 studies[6–9,11–15]. After completion of PNL, a nephrostomy tube was routinely placed in all including studies. A double-J stent was routinely placed in 5 studies[10–13,15].

**Table 2:** Variations in PCNL techniques, as stated in the methods section: An overview.

Studies	Imaging	Dilator			sheath size(Fr)	Lithotripsy technique				
		Balloon	Metal	Amplatz		Pneumatic	Ultrasonic	Laser	Postoperative US	NT(Fr)
AL-KOHLANY KM et al 2005					24	Y	Y			18
Aminsharifi A et al 2016	F			Y	30	Y				18
El-Nahas AR et al 2014	F			Y	24/30	Y				18
Falahatkar S et al 2009	F			Y	30	Y			Y	18
Zhang FBY et al 2017	US			Y	24	Y	Y			16
Cao GZ et al 2008	F/US			Y	18			Y	Y	14/16
Fei X et al 2012	US				24	Y	Y			12
liang TS et al 2010	US			Y	16/18	Y			Y	16/18
Yang X et al 2014	US		Y		24	Y	Y		Y	12
Zheng B et al 2011	F			Y	18	Y			Y	18

F = fluoroscopy, US = Ultrasound, NT = nephrostomy tube, US = ureteral stent, PCNL = Percutaneous nephrolithotomy, Y = yes

## Primary outcomes

### SFR

**Fig 2:**
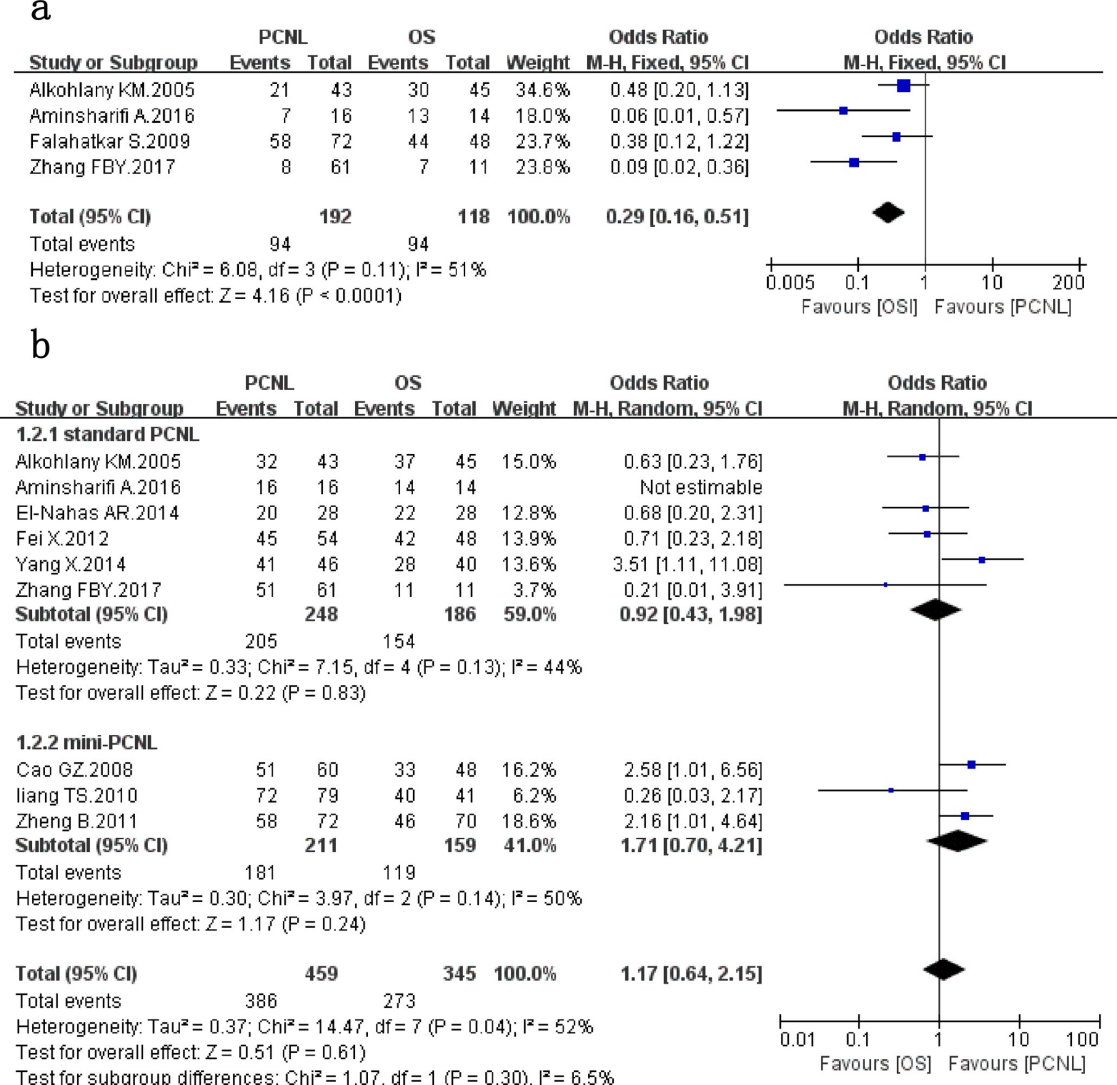
Forest plot and meta-analysis of immediate SFR(a) and final SFR(b).

Four studies[6–8,15] that assessed 310 patients reported on *immediate-SFR* (Fig 2A). PCNL provided a significantly lower immediate-SFR compared with OS (*OR*: 0.29; 95% *CI*: 0.16, 0.51; *P* < 0.0001), with no significant between-study heterogeneity (*χ*^*2*^ = 6.08, *df* = 3, *p* = 0.11, *I*^*2*^ = 51%).

Data on *final SFR* were available in 9 studies[6–14], which evaluated 804 patients(Fig 2B). Meta-analysis of the 9 studies indicated that there was no significant difference between the two groups (*OR*: 1.17; 95% *CI*: 0.64, 2.15; *p* = 0.61), with significant between-study heterogeneity (*χ*^*2*^ = 14.47, *df* = 7, *P* = 0.04, *I*^*2*^ = 52%). In subgroup analysis, the results of the subgroup of standard PCNL and mini-PCNL were consistent with the overall results, with the pooled OR values of 0.92(95%*CI*: 0.43, 1.98; *P* = 0.83) and 1.71(95%*CI*: 0.7, 4.21; *P* = 0.24), respectively. However, the between-study heterogeneity was significantly reduced in subgroup analysis.

### Complications

**Fig 3:**
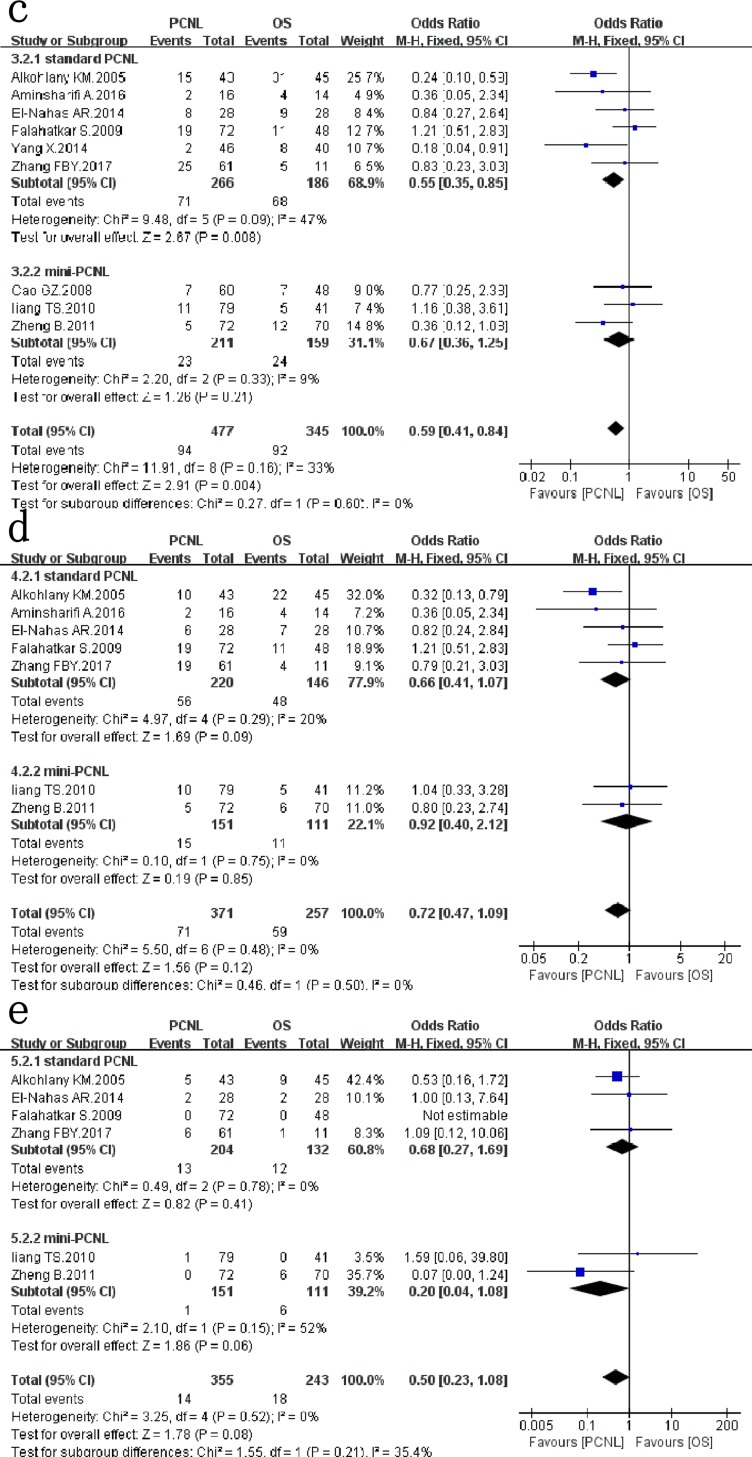
Forest plot and meta-analysis of overall complications(c), minor complications(d) and major complications(e).

Nine studies[6–12,14,15] that assessed 822 patients reported on *overall complications* (Fig 3C). PCNL provided significantly lower overall complications compared with OS (*OR*: 0.59; 95% *CI*: 0.41, 0.84; *P* = 0.004), with no significant between-study heterogeneity (*χ*^*2*^ = 11.91, *df* = 8, *p* = 0.16, *I*^*2*^ = 33%). In subgroup analysis, the result of the subgroup of standard PCNL was consistent with the overall results, with the pooled OR values of 0.55(95%*CI*: 0.35, 0.85; *P* = 0.008), but with moderate between-study heterogeneity (*χ*^*2*^ = 9.48, *df* = 5, *p* = 0.09, *I*^*2*^ = 47%). However, there was no significant difference between mini-PCNL and OS (*OR*: 0.67; 95% *CI*: 0.36, 1.25; *p* = 0.21), with no between-study heterogeneity (*χ*^*2*^ = 2.2, *df* = 2, *P* = 0.33, *I*^*2*^ = 9%).

Data on *minor complications (Clavien I-II)* were available in 7 studies[6–9,11,12,14,15], which evaluated 628 patients(Fig 3D). Meta-analysis of the 7 studies indicated that there was no significant difference between the two groups (*OR*: 0.72; 95% *CI*: 0.47, 1.09; *p* = 0.12), with no between-study heterogeneity (*χ*^*2*^ = 5.5, *df* = 6, *P* = 0.48, *I*^*2*^ = 0%). In subgroup analysis, the results of the subgroup of standard PCNL and mini-PCNL were consistent with the overall results, with no between-study heterogeneity respectively.

Six studies[6,7,9,11,14,15] reported *major complications(Clavien III-V)* (Fig 3E). There was no significant difference between the two groups (OR: 0.5; 95% CI: 0.23, 1.08; *P* = 0.08), with no between-study heterogeneity (*χ*^*2*^ = 3.25, *df* = 4, *P* = 0.52, *I*^*2*^ = 0%). In subgroup analysis, the results of the subgroup of standard PCNL and mini-PCNL were consistent with the overall results, with no between-study heterogeneity respectively.

## Secondary outcomes

### Operative times

**Fig 4:**
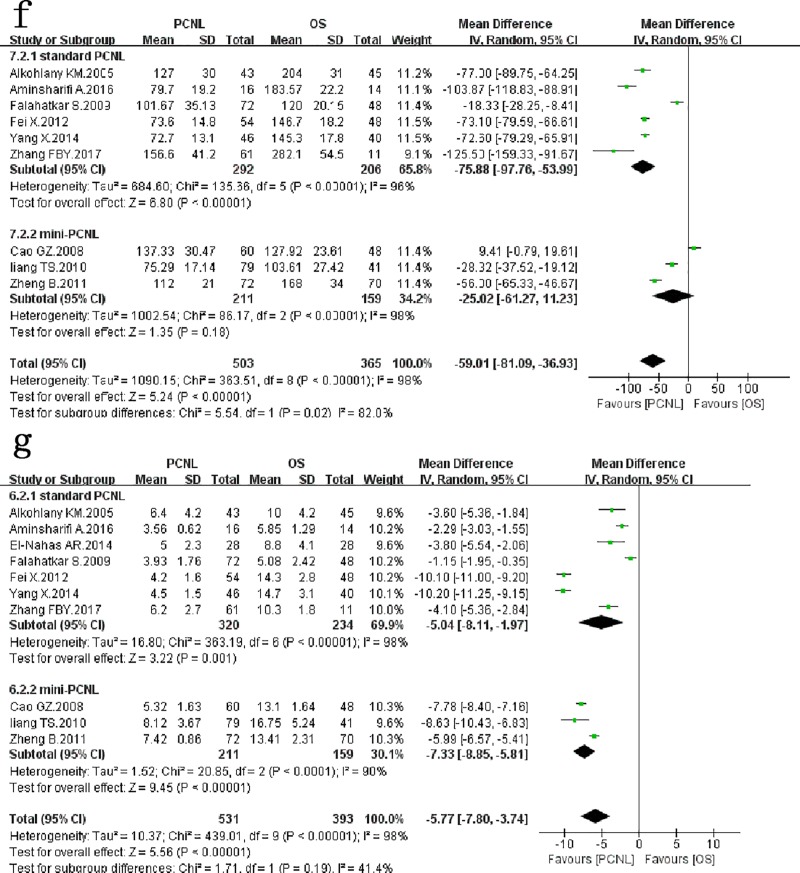
Forest plot and meta-analysis of operative times(f) and hospitalization times(g).

Nine studies[6–8,10–15] assessed 868 patients and reported on *operative times* (Fig 4F) between the two groups favouring the PCNL(*WMD*: -59.01min; 95% *CI*: -81.09, -36.93; *p* < 0.00001), with significant between-study heterogeneity (*χ*^*2*^ = 363.51, *df* = 8, *p* < 0.00001, *I*^*2*^ = 98%). In subgroup analysis, the results of the subgroup of standard PCNL were consistent with the overall results, with significant between-study heterogeneity. However, there was no significant difference between mini-PCNL and OS (*WMD*: -25.02min; 95% *CI*: -61.27, 11.23; *p* < 0.00001), with significant between-study heterogeneity.

### Hospitalization times

Ten studies[6–15] assessed 924 patients and reported on *hospitalization times* (Fig 4G) between the two groups favouring the PCNL(*WMD*: -5.77d; 95% *CI*: -7.80, -3.74; *p* < 0.00001), with significant between-study heterogeneity (*χ*^*2*^ = 439.01, *df* = 9, *p* < 0.00001, *I*^*2*^ = 98%). In subgroup analysis, the results of the subgroup of standard PCNL and mini-PCNL were consistent with the overall results, with significant between-study heterogeneity respectively.

### Blood loss

**Fig 5:**
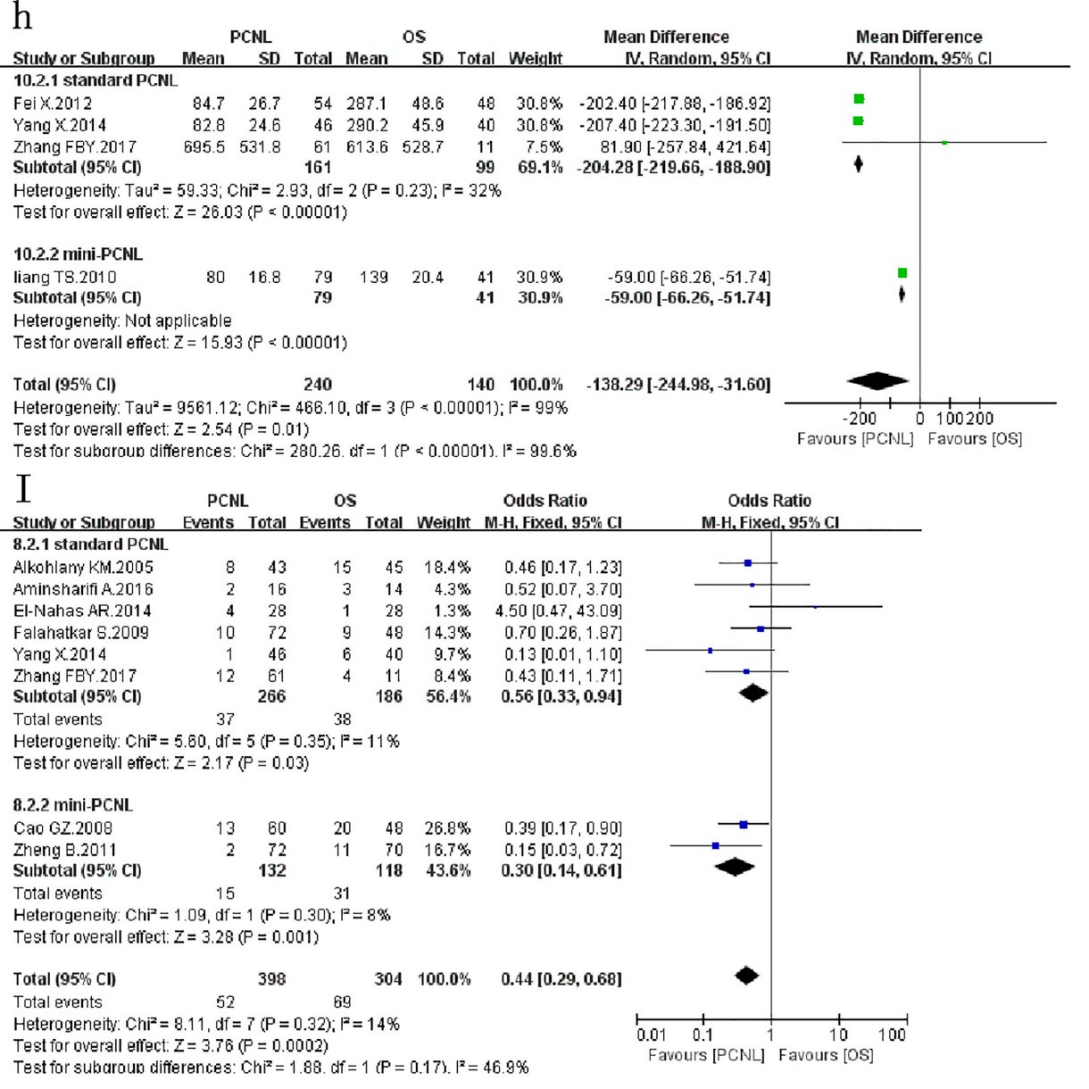
Forest plot and meta-analysis of blood loss(h) and blood transfusion(I).

Four studies[6,12–14] assessed 380 patients and reported on *blood loss* (Fig 5H) between the two groups favouring the PCNL(*WMD*: -138.29*ml*; 95% *CI*: -244.98, -31.6; *p* = 0.01), with significant between-study heterogeneity (*χ*^*2*^ = 466.1, *df* = 3, *p* < 0.00001, *I*^*2*^ = 99%). In subgroup analysis, the results of the subgroup of standard PCNL and mini-PCNL were consistent with the overall results, with no significant between-study heterogeneity.

### Blood transfusion

Data on *blood transfusion* were available in 8 studies[6–12,15], which evaluated 702 patients(Fig 5I). Meta-analysis indicated that PCNL provided less blood transfusion compared with OS (*OR*: 0.44; 95% *CI*: 0.29, 0.68; *P* = 0.00002), with no between-study heterogeneity (*χ*^*2*^ = 8.11, *df* = 7, *P* = 0.32, *I*^*2*^ = 14%). In subgroup analysis, the results of the subgroup of standard PCNL and mini-PCNL were consistent with the overall results.

### Sensitivity analysis

When high quality studies were assessed, no change in terms of the significance of each of the outcomes except for overall complications. Meta-analysis of 7 high quality studies[6–10,14,15] revealed that there was no significant difference between the two groups for surgical treatment of patients with staghorn stones, with no between-study heterogeneity. Between-study heterogeneity was significantly reduced by the sensitivity analysis for final SFR and blood loss. While heterogeneity remained statistically significant in operative times and hospitalization times. (Table 3)

**Table 3:** Sensitivity analysis according to high quality studies comparing PCNL and OS.

	Number	patients			WMD/OR (95% CI)	p value	Study heterogeneity			
	studies	PCNL	OS	Total			χ^*2*^	df	I^*2*^%	p-value
Final SFR	6	287	187	474	0.8(0.34,1.91)	0.62	7.79	4	49	0.1
Over complications	7	359	235	594	0.69(0.46,1.03)	0.07	8.51	6	29	0.2
Minor complications	6	299	187	486	0.71(0.45,1.1)	0.13	5.47	5	9	0.36
Major complications	5	283	173	456	0.73(0.31,1.74)	0.48	0.73	3	0	0.87
Operative times	6	331	207	538	-55.56(-89.95,-21.17)	0.002	236.26	5	98	<0.00001
Hospitalization times	7	359	235	594	-4.46(-6.86,-2.06)	0.0003	231.7	6	97	<0.00001
Blood loss	2	140	52	192	-58.94(-66.19,-51.68)	<0.00001	0.66	1	0	0.42
Blood transfusion	6	280	194	474	0.55(0.35,0.88)	0.01	4.49	5	0	0.48

WMD = weighted mean difference, OR = odds ratio, 95% CI = 95% confidence interval

### Publication bias outcomes

**Fig 6:**
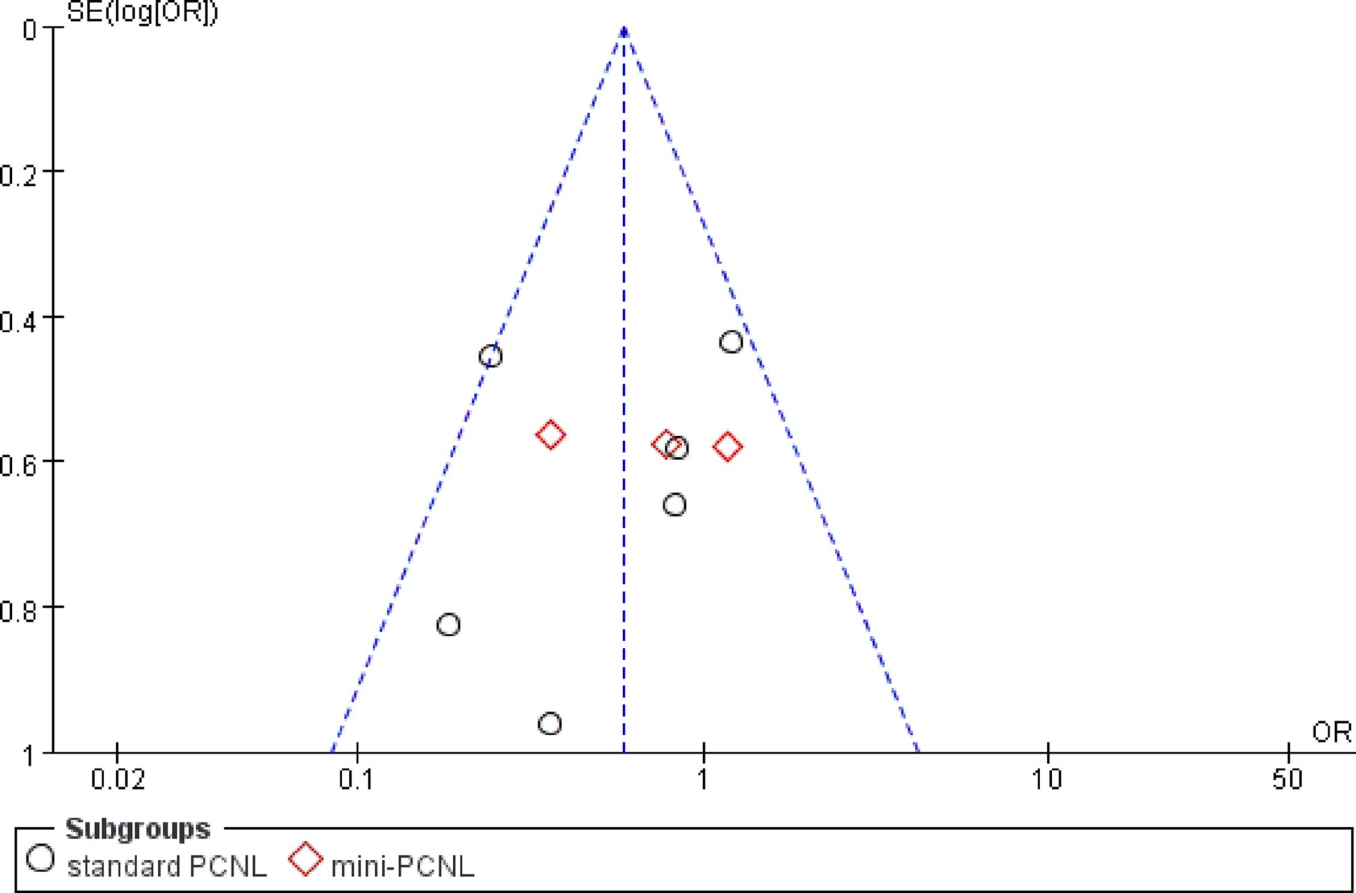
Funnel plot for assessing publication bias (overall complications).

Funnel plots were conducted to assess the publication bias in this meta-analysis that reported overall complications (Fig 6). All studies lie inside the 95% CIs, with an even distribution around the vertical, indicating no obvious publication bias.

## Discussion

This meta-analysis of 10 studies, which included 921 patients from 1 RCT, 2 prospective case-control studies and 7 retrospective case-control studies, comparing the efficacy and safety of PCNL and OS for patients with staghorn stones. The pooled data showed that there was no significant difference in final-SFR between PCNL and OS, while PCNL provided a significantly lower immediate-SFR compared with OS. PCNL provided significantly lower overall complication rate compared with OS. However, no significant differences were found in minor complications (Clavien I-II) and major complications (Clavien III-V). PCNL provided significantly shorter operative times and hospitalization times compared with OS. And PCNL provided significantly less blood loss and blood transfusion compared with OS. In subgroup analysis, there was no significant difference for overall complications and operative times between mini-PCNL and OS. In sensitivity analysis, there was no significant difference for overall complications between PCNL and OS.

In the application of urolithiasis surgery, SFR represents a paramount important parameter. Al-Kohlany KM et al.[7] also found that both PCNL and OS were comparable in regard to SFRs at discharge home and at follow-up. However, Zhang FBY et al.[6] found that OS provided a significantly higher final SFR compared with PCNL (97.5% vs 76.1, *p* < 0.001). Our pooled data showed that although OS provided a significantly higher immediate-SFR compared with PCNL, no significant difference was found in final-SFR. And the results of the subgroup and sensitivity analysis of standard PCNL and mini-PCNL were consistent with the overall results.

The safety of the patients is also an important parameter. Our pooled data showed that PCNL provided significantly lower overall complication rate compared with OS. And no significant differences were found in minor complications (Clavien I-II) and major complications (Clavien III-V). However, there was no significant difference for overall complications in subgroup analysis between mini-PCNL and OS. And no significant difference was found for overall complications between PCNL and OS in sensitivity analysis. Our pooled data also found that although PCNL provided significantly shorter operative times compared with OS, no significant difference was found for operative times between mini-PCNL and OS in subgroup analysis. The disadvantage of small instruments is that it is necessary to fragment staghorn stones into smaller pieces that fit through the narrower sheaths, which would increase the operative times of the mini-PCNL. Prolonged operating times lead the trend towards higher complications for mini-PCNL compared with standard PCNL.

A great deal of studies[12–14] found that PCNL provided significantly less blood loss compared with OS. While Zhang FBY et al.[6] found that OS provided less blood loss compared with OS, though with no statistically significant. However, our pooled data showed that PCNL provided significantly less blood loss and blood transfusion compared with OS. And the results of the subgroup and sensitivity analysis of standard PCNL and mini-PCNL were consistent with the overall results.

Several limitations of our meta-analysis should be taken into consideration when interpreting the results. First of all, nine of ten studies were non-RCTs. Those results should be interpreted with caution given the potential for selection and treatment bias due to the non-RCT heterogeneous nature. Heterogeneity among studies were found to be high for several parameters, including final SFR, operative times, hospitalization times, and blood loss. Although between-study heterogeneity was significantly reduced by the sensitivity analysis for final SFR and blood loss, heterogeneity of operative times and hospitalization times remained statistically significant. The differences in surgical technique and surgical experience, and outcome definitions have all acted an important role in the heterogeneity. The surgical technique for PCNL and OS, the diameters and location of stones were not similar across the different studies. Overall, only 10 studies with 921 patients could be included in the meta-analysis. Such a small number of studies were unable to make strong conclusion. Thus, further large sample prospective, multi-centric studies and RCTs should be undertaken to confirm our findings.

Nevertheless, our meta-analysis was conducted at an appropriate time. Ten data have accumulated for analysis. We applied multiple strategies to identify studies, strict criteria to include and evaluate the methodological quality of the studies, and subgroup analysis to minimize the heterogeneity. Hence, we provide the most up-to-date information in surgical treatment of patients with staghorn calculi, and this could guide urologists and patients to decide on the surgical method, and to select the optimal therapy.

## Conclusion

Our systematic review and meta-analysis demonstrated that standard PCNL seems to be a safe and feasible alternative compared to OS or mini-PCNL for patients with staghorn stones with many advantages, such as shorter hospitalization times and operative times, less blood loss and blood transfusion, and without increasing complications nor decreasing final SFR. However, our conclusion should be treated prudently and further large sample prospective, multi-centric studies and randomized control trials should be undertaken to confirm our findings.

## Supporting information

S1 Checklist PRISMA checklist. (DOC)

## Abbreviations

PCNL: Percutaneous Nephrolithotomy
OS: open surgery
WMD: Weighted mean difference
OR: Odds ratio
95%CI: 95% confidence interval
LE: Level of evidence
RCT: Randomized controlled trial
SFR: stones free rate
